# Implicit Recognition of Familiar and Unfamiliar Faces in Schizophrenia: A Study of the Skin Conductance Response in Familiarity Disorders

**DOI:** 10.3389/fpsyt.2017.00181

**Published:** 2017-09-28

**Authors:** Aurely Ameller, Aline Picard, Fabien D’Hondt, Guillaume Vaiva, Pierre Thomas, Delphine Pins

**Affiliations:** ^1^Laboratoire de Sciences Cognitives et Sciences Affectives (SCALab), CNRS UMR 9193, University of Lille, Lille, France; ^2^CURE, Clinique de Psychiatrie, CHU Lille, Lille, France

**Keywords:** schizophrenia, skin conductance response, familiarity disorders, Capgras, Fregoli

## Abstract

**Objective:**

Familiarity is a subjective sensation that contributes to person recognition. This process is described as an emotion-based memory-trace of previous meetings and could be disrupted in schizophrenia. Consequently, familiarity disorders could be involved in the impaired social interactions observed in patients with schizophrenia. Previous studies have primarily focused on famous people recognition. Our aim was to identify underlying features, such as emotional disturbances, that may contribute to familiarity disorders in schizophrenia. We hypothesize that patients with familiarity disorders will exhibit a lack of familiarity that could be detected by a flattened skin conductance response (SCR).

**Method:**

The SCR was recorded to test the hypothesis that emotional reactivity disturbances occur in patients with schizophrenia during the categorization of specific familiar, famous and unknown faces as male or female. Forty-eight subjects were divided into the following 3 matched groups with 16 subjects per group: control subjects, schizophrenic people with familiarity disorder, and schizophrenic people without familiarity disorders.

**Results:**

Emotional arousal is reflected by the skin conductance measures. The control subjects and the patients without familiarity disorders experienced a differential emotional response to the specific familiar faces compared with that to the unknown faces. Nevertheless, overall, the schizophrenic patients without familiarity disorders showed a weaker response across conditions compared with the control subjects. In contrast, the patients with familiarity disorders did not show any significant differences in their emotional response to the faces, regardless of the condition.

**Conclusion:**

Only patients with familiarity disorders fail to exhibit a difference in emotional response between familiar and non-familiar faces. These patients likely emotionally process familiar faces similarly to unknown faces. Hence, the lower feelings of familiarity in schizophrenia may be a premise enabling the emergence of familiarity disorders.

## Introduction

Misidentification disorders refer to disorders in a patient’s sense of personal relatedness to other people; patients deny the identity of other people who are either close to them or are strangers (1). Experiences in which patients do not recognize someone they know or believe that someone they know has been replaced by another person to persecute them are core features of misidentification disorders. In Fregoli syndrome (2), patients recognize unknown people and believe that they are friends or relatives who have assumed the faces of strangers. These patients often have persecutory delusions centered upon these relatives or friends. The most commonly described misidentification disorder is Capgras syndrome (3, 4). Patients with Capgras syndrome recognize specific familiar faces but have the delusional belief that the familiar person is an impostor or a clone appearing as whom they claim to be. Patients with CS argue that there is something “weird” in the personality of the person and/or that minor physical features (e.g., iris color) have changed.

The following two independent but cooperative processes are beneficial for accessing information regarding an individual person: identification and recognition (5). Identification is defined as the retrieval of the identity of an object or individual. Recognition is the knowledge that an object or an individual has been previously encountered. Recognition is a cognitive process is that associated with memory. Recognition can be further divided into the following two different processes (5): recollection and familiarity. Recollection refers to the conscious remembrance of a prior experience, which provides access to the contextual details of this prior event (e.g., where when). Familiarity is a subjective sensation that occurs during stimulus processing and is unconsciously attributed to past experiences. Hence, familiarity is a preconscious process that contributes to the recognition of an individual without the need to consciously remember the context in which the person has been previously met. Familiarity has been described as an emotion-based memory-trace of previous meetings and acts as a facilitator (6). The more frequently a person encounters another, the easier it is to recognize him or her. Hence, misidentification disorder may, in fact, be a core defect in familiarity. Therefore, the ambiguous name “misidentification” should be reconsidered. In this article, we use the term “familiarity disorders,” which is a more accurate term than “misidentification disorders,” based on the neurocognitive processes described above.

Familiarity disorders can be features of many psychiatric and neurological disorders (such as Alzheimer’s or Lewy dementia, ischemic stroke, and Parkinson’s disease). In particular, familiarity disorders are widely described in patients in schizophrenia, and these disorders may be the cause of the core defects in social interactions. Therefore, a better understanding of familiarity disorders in schizophrenia is critical. Familiarity disorders encompass two polarities. Hypofamiliarity refers to a lack of familiarity of individuals close to the person (such as in Capgras syndrome), whereas hyperfamiliarity refers to an increased sense of familiarity of unknown people (such as in Fregoli syndrome). Nevertheless, the distinction between hypo- and hyperfamiliarity appears to be weak because the co-occurrence of Capgras syndrome and Fregoli syndrome has been described in some schizophrenic patients (7, 8). Indeed, patients may first present with Capgras syndrome and then develop Fregoli syndrome in the course of their disease (9). Both syndromes might represent different disturbances in the same system that can oscillate and, therefore, allow both syndromes to be present in the same patient. Indeed, patients may show certain symptoms of familiarity disorders regardless of their polarity (hyper- or hypofamiliarity), which further supports the hypotheses that these syndromes are on a continuum.

The familiarity evoked by a particular stimulus elicits an overt emotional response that is mediated by autonomic arousal (10, 11). An easily measurable index of this autonomic arousal is the skin conductance response (SCR). Indeed, the SCR is a reliable autonomic marker of central activation that is indicative of emotional arousal and its somatovisceral impact (12). SCRs have been recorded in control subjects and patients with schizophrenia with and without familiarity disorders, who were passively looking at pictures of famous and unknown faces (13). While the control subjects and the patients without familiarity disorders displayed larger SCRs in response to the famous faces than that to the unknown faces, the patients with Capgras syndrome did not show a difference in the SCR to both types of faces. Patients with familiarity disorders appear to consider the famous faces unfamiliar. However, the sample size was too small for a definitive conclusion; only five patients with Capgras syndrome were tested. Moreover, Capgras syndrome is described as affecting the recognition of close people who are affectively related to the patient (2–4). Furthermore, famous people are not always associated with a strong emotional investment (14).

A model proposed by Hirstein (1) in the field of theory-of-mind posits that familiar people are represented by both allocentric and egocentric representations. Allocentric representations are established during each encounter with a person and stored in semantic memory in the third-person perspective. In contrast, egocentric representations are simulated representations of a person in the first-person perspective. General representations, which are established by the aggregation of autobiographical memory information into concepts about people or objects, are activated for strangers, allowing interactions with unknown people by simulating their mental state. According to Hirstein, prior to affective failure, patients with Capgras syndrome are unable to access the egocentric representation of a relative. This inability may create a feeling of ambiguity regarding the person’s familiarity because the two representations do not match, and the activation of a generic representation is elicited as it would be in the presence of strangers.

In the present study, we characterized the affective processing involved in familiarity disorders in patients with schizophrenia. We hypothesized that the familiarity disorders in schizophrenia are caused by emotional disturbances. Therefore, the SCR was recorded during a face categorization task using a set of specific highly familiar and homogeneous faces selected from the subjects’ set of specific familiar individuals. Indeed, the subjects’ degree of familiarity with these faces was both quantitatively and qualitatively more important and controlled than that with famous faces or recently learned faces of unfamiliar people. As previously suggested, hypo- and hyperfamiliarity might result from failures in a single system. Consequently, we hypothesized that in contrast to the controls and patients without familiarity disorders (FD−), the amplitude of the affective response in patients with familiarity disorders (FD+) would not be different regardless of the polarity of their disorder and the familiarity of the faces.

## Materials and Methods

### Participants

Thirty-eight outpatients with schizophrenia (nine women) but without any psychiatric comorbidity (DSM-IV criteria) were recruited at the Lille University Hospital. The patients were assessed and assigned to one of two different groups based on their clinical relevance. FD+ patients showed at least one symptom of familiarity disorders. FD− patients had no history of such symptoms. The familiarity disorders were assessed using eight questions regarding hypo- and hyperfamiliarity experiences. The following four questions were used to assess hypofamiliarity: have you ever not recognized a familiar person? Have you ever experienced a feeling of strangeness or oddity in the presence of a familiar person? Had this familiar person changed? Was it a double? The following four questions were used to assess hyperfamiliarity: Have you ever been fooled into believing a stranger was a familiar person? Have you ever experienced a sense of familiarity to a stranger? Have you ever been convinced that you recognize a familiar person in someone unknown? Did that person appear to be a double? The FD+ group included eight patients with hypofamiliarity symptoms, five patients with hyperfamiliarity symptoms and three patients with both hyper- and hypofamiliarity symptoms. The FD+ patients showed at least one symptom of FD. All patients in the FD+ group reported that they experienced an FD at the time of the experiment. No patient in the FD− group had ever experienced such symptoms.

Eighteen healthy controls who matched the patients were recruited *via* local advertising. The control subjects had no psychiatric disorders as assessed by the MINI (15). There was no clinical evidence of prosopagnosia in any participant. No participant had an active substance use disorder based on a clinical interview. Due to the lack of SCR recordings of 6 patients and 2 controls, 32 patients with schizophrenia (9 left-handed) and sixteen healthy controls (3 left-handed) completed the entire study. Such an absence of recording has been previously described in the SCR literature [for review, see Ref. (16)]. The patients were divided into the following two groups: people with schizophrenia without FD [FD− (*N* = 16)] and people with schizophrenia with FDs [FD+ (*N* = 16)].

The participants’ characteristics are summarized in Table 1. This study was approved by the local ethics committee (CPP, Nord-Ouest IV, France), and written informed consent was obtained from each participant or guardian. All participants had normal or corrected-to-normal vision.

**Table 1:** Demographic data of the three groups of control subjects (controls), schizophrenic patients without familiarity disorders (FD−) and schizophrenic patients with familiarity disorders (FD+).

	Controls (*N* = 16)	FD− (*N* = 16)	FD+ (*N* = 16)	*P*
Age (years)	37.2 ± 10.7	37.2 ± 10.7	33.3 ± 9.6	0.66
Sex	9♂ 7♀	15♂ 1♀	12♂ 4♀	0.058
PANSS+		18.3 ± 3.3	22.2 ± 3.9	0.059
PANSS−		18.17 ± 2.9	21.18 ± 4.1	0.14
Diazepam equivalent		4.5 ± 3.1	5.7 ± 6.2	1.0
Chlorpromazine Equivalent (mg)		1033.4 ± 1117.5	857.3 ± 526.5	0.69

*The three groups described in the table are the control subjects (controls), schizophrenic patients without familiarity disorders (FD−), and schizophrenic patients with familiarity disorders (FD+)*.

*PANSS, Positive And Negative Symptoms Scale*.

### Procedures

**Figure 1:**
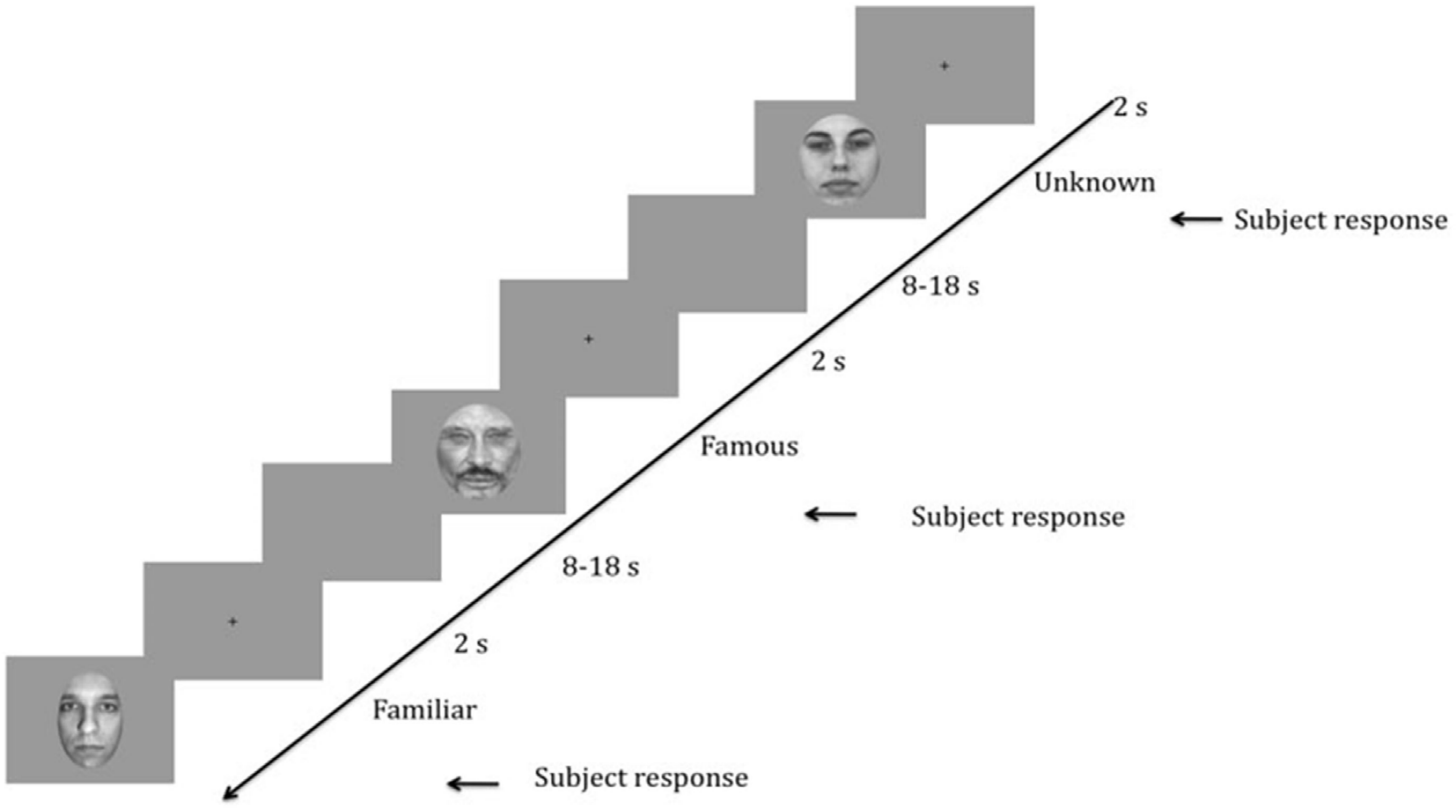
Schematic representation of the task. Time is presented in decreasing order from right to left. After the presentation of a fixation cross for 2 s, a face (randomly unknown, famous, or familiar) was presented to the participant until he/she responded. Then, a blank screen appeared for 8 to 18 s to allow time for the skin conductance response (SCR) recording. The random duration of the blank screen was to avoid SCR habituation effects.

The experimental procedures included two phases. The entire task required approximately 15 min to complete. During the first phase (see Figure 1), the SCR data were collected during the presentation of 30 different black and white photographs of faces (10 specific familiar, 10 famous, and 10 unknown), which were presented in a random order using E-Prime 2.0 software (Psychology Software Tools, Pittsburgh, PA, USA). The famous faces were selected from the Internet, and the unknown faces were selected from the neutral faces in the Karolinska Directed Emotional Faces (17, 18). All faces had neutral expressions (mouth closed) and a direct gaze direction and were presented once on a uniform gray background (800 × 600 pixels). The external features of the faces were removed, and the faces were equalized in size (300 × 462 pixels—11 × 9.5° degree of visual angle), brightness and contrast using Adobe Photoshop^®^. The participants placed their heads on a chin rest with their eyes positioned centrally 60 cm from the monitor (Intel computer, Sony screen, resolution 1,280 × 1,024 pixels, refresh rate 60 Hz). The participants were asked to categorize the face displayed as a male or female by pressing one of two keys on a keyboard as quickly as possible. An implicit recognition task was preferred to avoid having patients focus on face recognition while ensuring that their attention was directed toward the faces.

Because the frequency of encounters may influence familiarity processing, strict temporal criteria were applied in selecting the familiar persons for each participant. The familiar persons were colleagues or friends who were encountered several days a week for at least 6 months. For the patients, the familiar persons could have been selected (if needed) from among the medical staff using the same temporal criteria.

The homogeneity of the stimuli was assessed in the three familiarity conditions. Twenty healthy controls (10 males) were asked to rate the attractiveness and approachability of the specific familiar, famous and unknown faces from 1 to 10. As expected (19), the famous faces were rated higher in attractiveness than the specific familiar faces (*Z* = −3.84; *P* = 0.0001) and unknown faces (*Z* = −3.75; *P* = 0.0001), but there was no significant difference between the specific familiar and unknown faces (*Z* = −0.38; *P* = 0.70). Furthermore, there were no differences in approachability among the conditions (between famous and specific familiar faces: *Z* = −0.67; *P* = 0.49; between unknown and specific familiar faces: *Z* = −0.30; *P* = 0.76 and between unknown and famous faces: *Z* = −0.69; *P* = 0.48).

During the second phase, we ensured that all participants recognized the specific familiar and famous faces and did not recognize the unknown people. The participants were presented with the same pictures as those presented during phase one. The participants were asked to state the name of each person they recognized. No SCR data were recorded during phase 2.

### Data Recording and Analyses

#### Behavioral Data

During both tasks, the response accuracy and response time were recorded for each stimulus. The percentage of correct responses was calculated for each condition.

### SCR Data

The SCR data were recorded using the constant-voltage method (0.5 V) at a sampling rate of 600 Hz using a commercial skin conductance sampling device (BiopacMP35, Biopac Systems Inc., Goleta, Canada). Ag-AgCl electrodes (8 mm diameter active area) filled with 0.05 M NaCl electrolytes were attached to the palm side of the middle phalanges of the second and third fingers of the participants’ left hands. To avoid movement that could impair the SCR recording, the right fingers pressing the keys were comfortably positioned on the keyboard to limit the movement amplitude. To minimize the number of non-responders, prior to the experiment, we ensure that the SCR was elicited by mental calculation and deep breathing. The amplitude and latency of the SCR were measured.

The SCR data were then extracted using BSL-pro software©. The SCRs were determined using the standard latency criterion of 1–4 s (11, 16, 20, 21). The first peak in amplitude within this latency window was recorded. All SCR data were low-pass filtered at 50 Hz. Trials during which the stimulation did not produce an SCR were included in the mean data for each participant as a zero value. The amplitude of the SCR was defined as the difference in the SCR measurement between the peak and inflection curve after a stimulus presentation. To normalize the data, the amplitude of the SCR was logarithmically transformed [log(1 + amplitude)] (16, 20).

### Statistical Analyses

Using a repeated-measures ANCOVA to accommodate non-sphericity, a Greenhouse-Geisser correction was applied to the average SCR amplitude [log(1 + amplitude)] or behavioral data (reaction times and correct responses) using the three participant groups as the between-subject factor and the familiarity conditions as the within-subject factor. We used sex and age as cofactors. The polarity of the FD in the FD+ group was not examined due to a potential lack of statistical power. If the ANCOVA was significant, we conducted intragroup and intergroup comparisons using Student’s *t*-tests. The normality of the data (Levene’s test) was confirmed prior to performing a parametric test. Spearman’s correlations were performed in the patients to determine the correlations between the SCR amplitude in each condition and age, treatment (both chlorpromazine- and diazepam-equivalents) and symptom severity (PANSS subscores: positive or negative). All statistical analyses were performed using SPSS^®^ 15.0, and the level of significance was set at *P* < 0.05. Because multiple comparisons were performed, we corrected the level of significance to *P* < 0.01 in the *post hoc* analyses (22).

## Results

### Demographic Data

The age, sex, positive and negative PANSS subscores, illness duration, and both chlorpromazine-equivalent and diazepam-equivalent doses were recorded for all participants or patients only according to their relevance (cf. Table 1). The three groups did not differ in age, while sex showed a trend toward significance. The negative symptom severity and illness duration did not differ between the patient groups; nevertheless, a trend toward significance was observed for the positive symptoms between both patient groups. All patients received second-generation antipsychotic medication with similar mean chlorpromazine-equivalent doses between the patient groups. Some patients received benzodiazepines (*N* = 4 in FD−; *N* = 7 in FD+) with similar mean diazepam-equivalent doses.

### Behavioral Data

During the first phase of the study, the categorization performance (male or female) was not different between the groups (see Table 2 for more details). There was no significant group effect on accuracy [*F*_(2,45)_ = 0.03; *P* = 0.96] or a familiarity condition effect [*F*_(2,90)_ = 2.16; *P* = 0.12]. No group × familiarity condition interaction was observed [*F*_(4,90)_ = 0.41; *P* = 0.80]. The response times were not significantly different between the groups [*F*_(2,45)_ = 3.45; *P* = 0.53] or among the familiarity conditions [*F*_(2,90)_ = 0.08; *P* = 0.91], and there was no group × familiarity condition interaction [*F*_(4,90)_ = 0.73; *P* = 0.57].

**Table 2:** Categorization of percentage of correct responses and response time in ms during phase 1 and phase 2 in the 3 groups of control subjects (controls), schizophrenic patients without familiarity disorders (FD−) and schizophrenic patients with familiarity disorders (FD+).

	Controls (*N* = 16)	FD− (*N* = 16)	FD+ (*N* = 16)
**Phase 1**			
Familiar categorization	92 ± 8	92 ± 10	91 ± 7
Familiar response time	1,051 ± 338	1,517 ± 536	1,423 ± 454
Famous categorization	96 ± 7	95 ± 7	94 ± 9
Famous response time	915 ± 327	1,517 ± 571	1,520 ± 703
Unknown categorization	95 ± 5	94 ± 12	97 ± 6
Unknown response time	1,004 ± 334	1,517 ± 833	1,496 ± 455
**Phase 2**			
Familiar categorization	92 ± 8	92 ± 10	91 ± 7
Famous categorization	96 ± 7	95 ± 7	94 ± 9
Unknown categorization	95 ± 5	94 ± 12	97 ± 6

During the second phase of the study, the recognition performance did not show a significant group effect on accuracy [*F*_(2,45)_ = 2.90; *P* = 0.65] or a familiarity condition effect [*F*_(2,90)_ = 0.95; *P* = 0.38]. There was no group × familiarity condition interaction [*F*_(4,90)_ = 0.69; *P* = 0.59].

### SCR Data

#### Main Results

**Figure 2:**
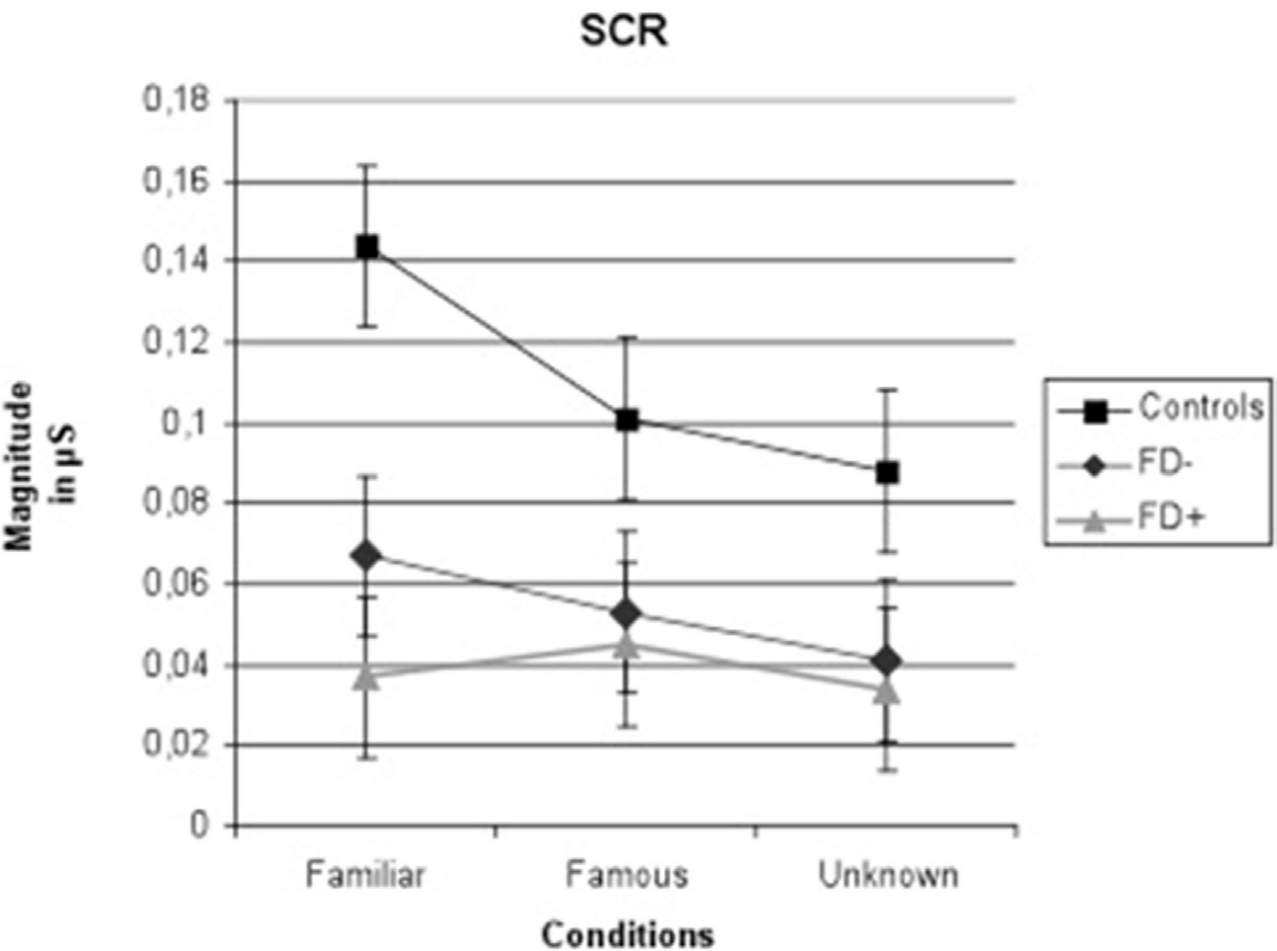
Amplitude of skin conductance response (SCR) in μS (microsiemens) in the three groups of control subjects (controls), schizophrenic patients without familiarity disorders (FD−), and schizophrenic patients with familiarity disorders (FD+) in the three conditions. Error bars represent the standard deviation.

**Figure 3:**
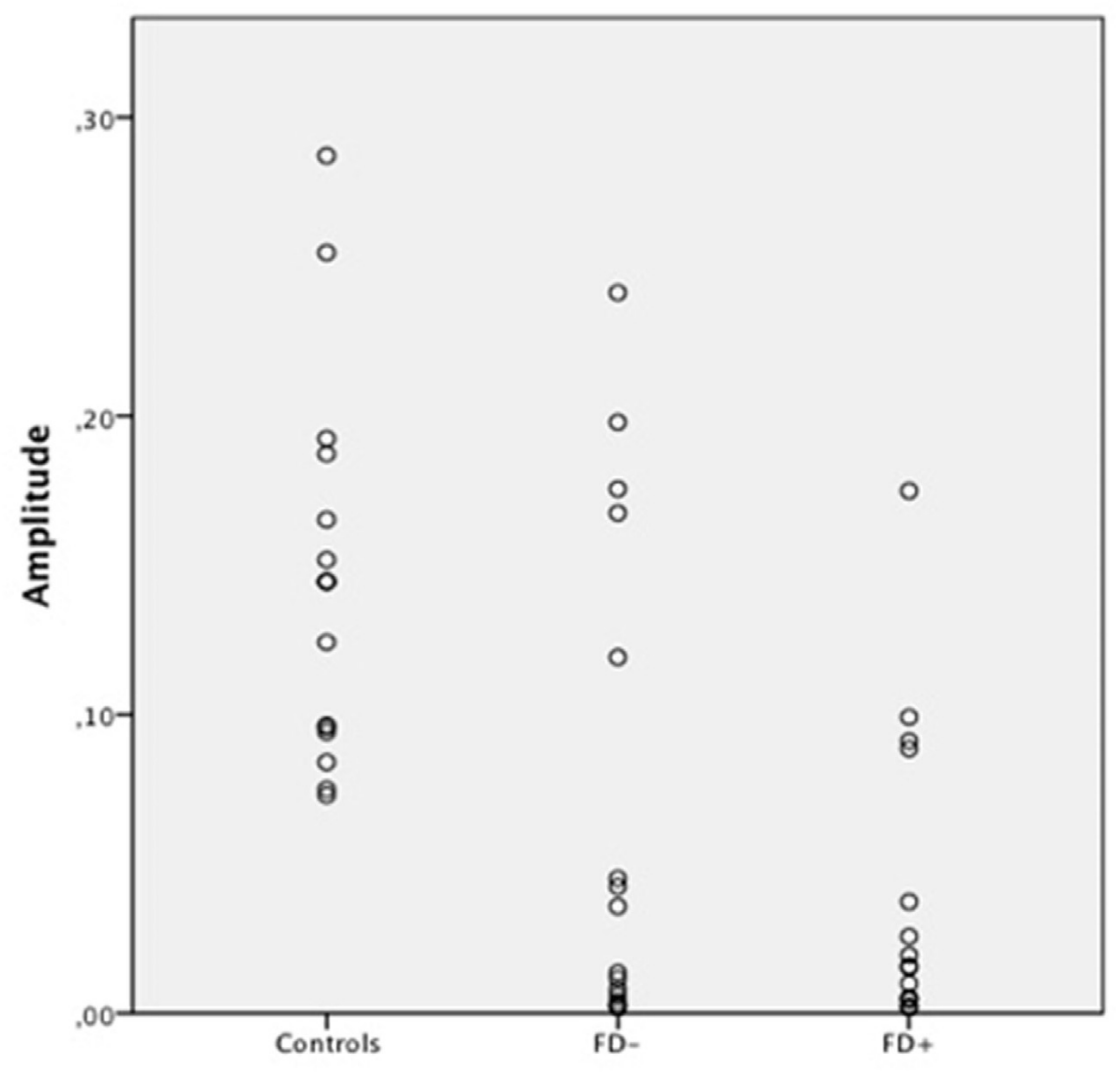
Scatter Plot of the amplitude of skin conductance response (SCR) in μS (microsiemens) in the three groups of control subjects (controls), schizophrenic patients without familiarity disorders (FD−), and schizophrenic patients with familiarity disorders (FD+) in the familiar conditions.

**Figure 4:**
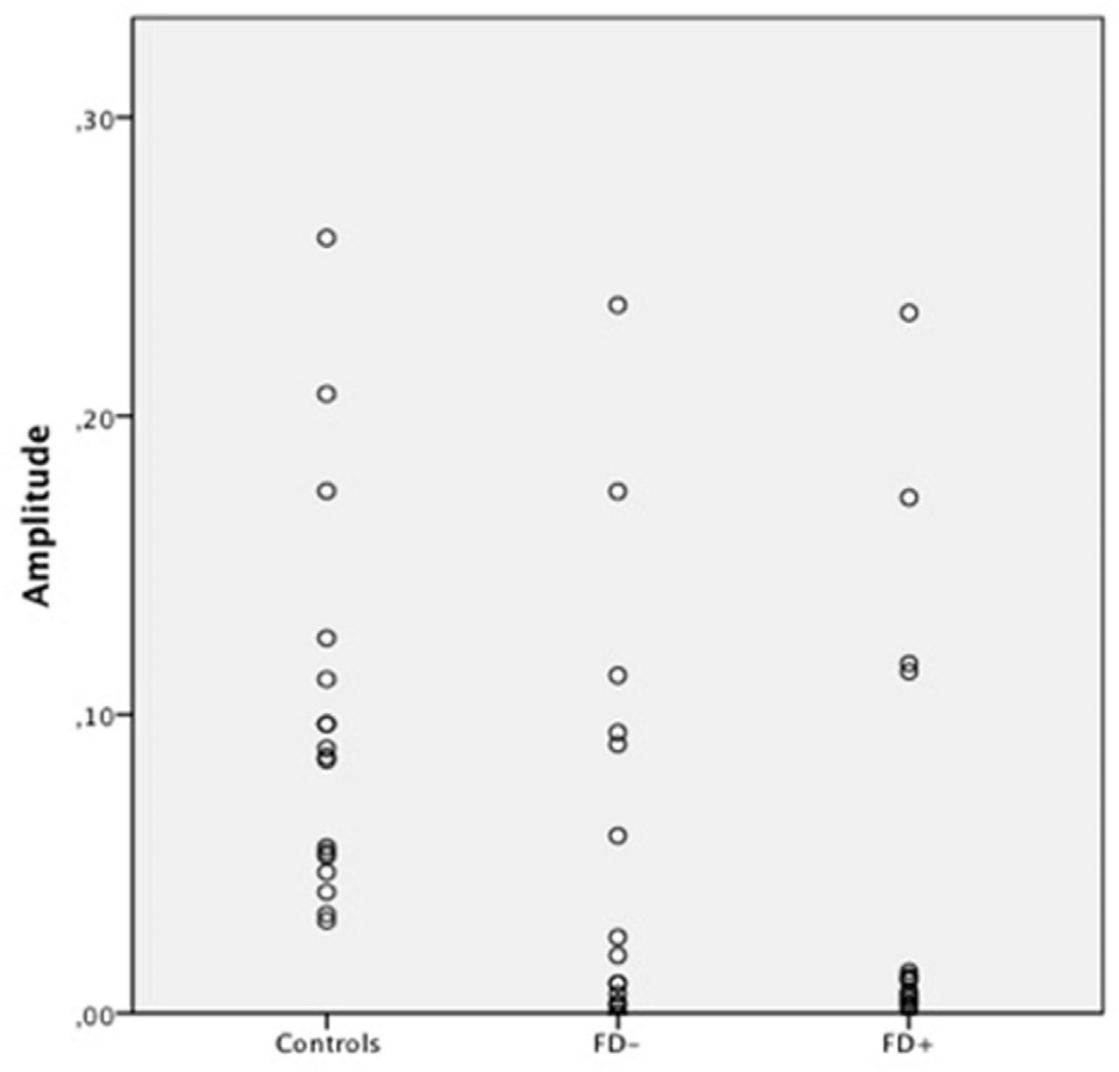
Scatter Plot of the amplitude of skin conductance response (SCR) in μS (microsiemens) in the three groups of control subjects (controls), schizophrenic patients without familiarity disorders (FD−), and schizophrenic patients with familiarity disorders (FD+) in the famous conditions.

**Figure 5:**
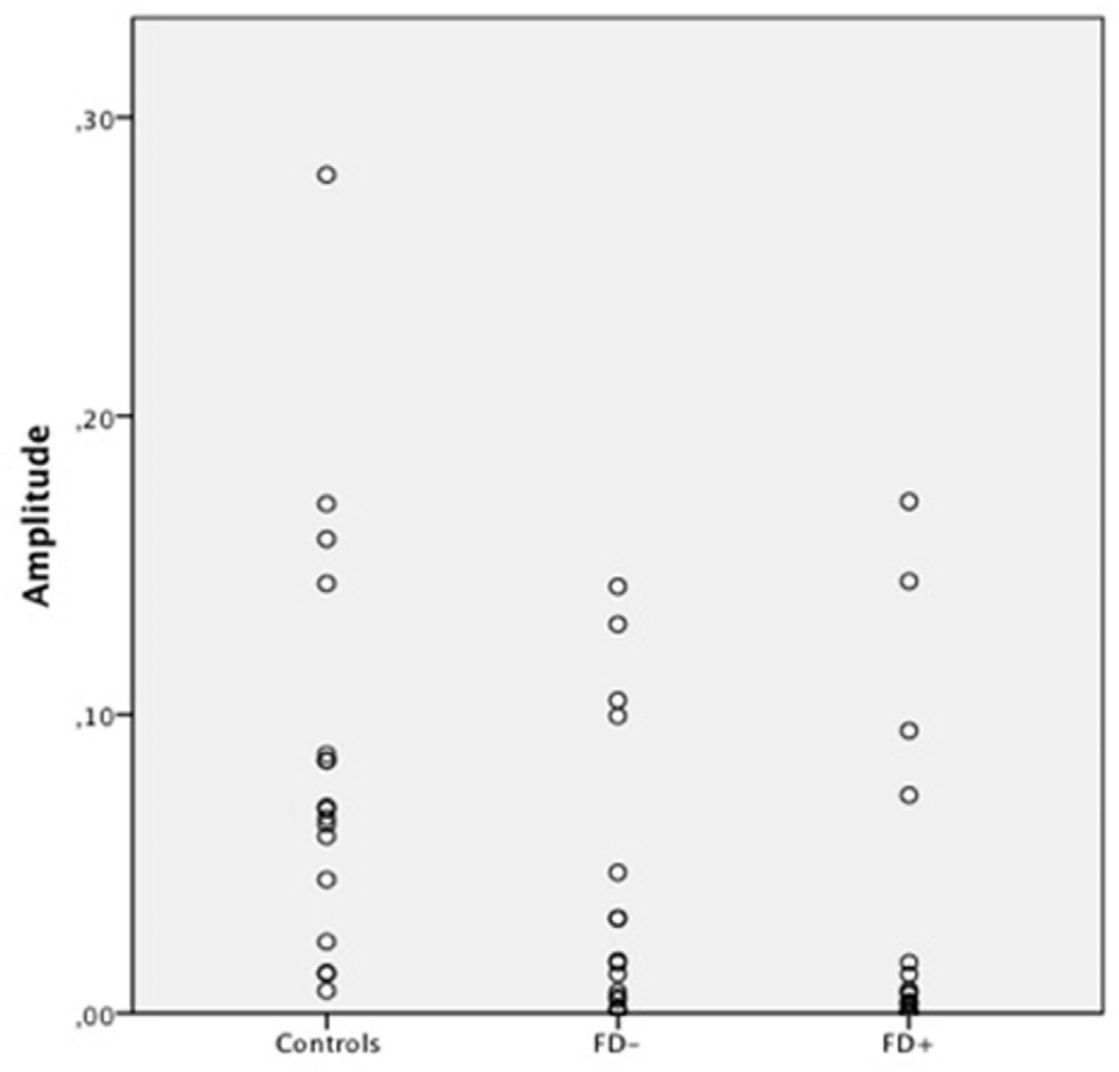
Scatter Plot of the amplitude of skin conductance response (SCR) in μS (microsiemens) in the three groups of control subjects (controls), schizophrenic patients without familiarity disorders (FD−), and schizophrenic patients with familiarity disorders (FD+) in the unknown conditions.

The results of the ANCOVA showed significant effects of familiarity conditions [*F*_(2,90)_ = 4.82; *P* = 0.003; η^2^ = 0.28] and group [*F*_(2,45)_ = 6.09; *P* = 0.005; η^2^ = 0.21] on the SCR amplitude, and a significant group × condition interaction was observed [*F*_(4,90)_ = 4.82; *P* = 0.003; η^2^ = 0.28]. The results are presented in Figures 2–5.

A significant effect of familiarity conditions on the SCR amplitude was found in the *control group* [*F*_(2,30)_ = 30.99; *P* < 0.0001; η^2^ = 0.67]. The results showed higher SCRs in response to specific familiar faces (0.144 ± 0.062 μS) than those in response to famous faces (0.101 ± 0.063 μS), which, in turn, were higher than those in response to unknown faces [0.088 ± 0.070 μS; specific familiar vs. famous: *t*_(15)_ = 5.70; *P* < 0.0001, specific familiar vs. unknown: *t*_(15)_ = 6.80; *P* < 0.0001, famous vs. unknown: *t*_(15)_ = 2.01; *P* = 0.06]. The more familiar the faces, the higher the SCR.

In the *FD− patient group*, there was a trend toward an effect of familiarity condition on the SCR amplitude [*F*_(2,30)_ = 20.96; *P* = 0.07; η^2^ = 0.16]. The same amplitude profile as that in the controls was found in this subject group with decreasing SCRs from specific familiar faces (0.067 ± 0.083 μS) to unknown faces (0.04 ± 0.049 μS) and famous faces in an intermediate position (0.053 ± 0.071 μS). Nevertheless, the only significant effect was found between the specific familiar and unknown faces [*t*_(15)_ = 2.82; *P* = 0.01]. Thus, for the FD− patients, similarly to the controls, the more familiar the faces, the higher the SCR.

In the *FD* ± *patient group*, the global effect of familiarity on the SCR amplitude was not significant [*F*_(2,30)_ = 1.12; *P* = 0.33; η^2^ = 0.01], and no significant effect was found among the conditions. The SCR amplitude did not differ among the specific familiar faces (0.037 ± 0.049 μS), famous faces (0.045 ± 0.072 μS), and unknown faces (0.034 ± 0.053 μS). For the FD+ patients, the SCR amplitude did not differ regardless of the degree of the familiarity of the faces.

#### Groups of Interest

The SCR amplitude in the FD− patients was generally weaker in all conditions compared with the SCR in the control subjects, resulting in a significant group effect [*F*_(1,30)_ = 6.47; *P* = 0.01; η^2^ = 0.178]. Moreover, the comparison of the controls and FD− groups showed a trend toward a significant group × familiarity condition interaction [*F*_(2,60)_ = 3.45; *P* = 0.03; η^2^ = 0.10].

Regarding the FD− patients, the SCR amplitude in the FD+ patients was globally weaker than the SCR in the control subjects, resulting in a significant group effect [*F*_(1,30)_ = 11.44; *P* = 0.002; η^2^ = 0.27]. Moreover, the comparison of the control and FD+ groups showed a significant group × familiarity condition interaction [*F*_(2,60)_ = 16.18; *P* < 0.0001; η^2^ = 0.35]. Finally, the FD+ patients differed from the FD− patients only in the specific familiar faces condition [*t*_(30)_ = 6.72; *P* = 0.01].

#### Correlations

No significant correlations were found between the SCR amplitudes in response to the three familiarity conditions and age, treatment (both chlorpromazine-equivalents and diazepam-equivalents), and symptom severity (PANSS subscores).

#### Anticholinergic Power

Subsequently, to ensure that the anticholinergic effects of the medications did not affect the SCR data, all patient medications were classified as one of four degrees of potential anticholinergic power (three for “high potential” to zero for “no anticholinergic effect scientifically proven”) in accordance with a review initially conducted for geriatric use of anticholinergic properties (23). Then, we ensured that both patient groups did not differ in the repartition of the anticholinergic power (Chi2_(1)_ = 1.59; *P* = 0.29).

## Discussion

In the current study, we explored FDs in patients with schizophrenia using a three-condition task that enabled us to discriminate the SCRs to specific familiar, famous, and unknown faces. Our results demonstrated that in healthy subjects, the more familiar the person, the stronger the emotional response linked to the feeling of familiarity. The same process occurs in the FD− patients, although these patients display a weaker emotional response across all conditions as indicated by the lower SCR amplitudes. However, the FD+ patients experienced an absence of emotional responses linked to the feeling of familiarity regardless of the familiarity of the faces that were presented (known or unknown). The feeling of familiarity elicited by emotional processes is not modulated by the degree of familiarity of the perceived faces. This result cannot be attributed to a lack of face recognition, which could be specific to the FD+ patients, because our results suggest that schizophrenic patients do correctly recognize faces, at least based on the visual features of the pictures. The gradient of the SCR does not, in fact, reflect cognitive or perceptual processes but only emotional responses to faces. Our results are consistent with those found in previous studies in healthy individuals (13, 19, 24). These previous studies using SCR measurements have shown that control subjects have a greater emotional response to familiar people than that to strangers.

### Famous Faces Are Not Familiar Faces

Our study addressed the crucial question of whether FDs in patients with schizophrenia are more apparent with individuals close to the person considering the well-documented clinical description of this delusion (2–4). To the best of our knowledge, the present study is the first to investigate the SCR properties of FDs in patients with schizophrenia with respect to familiarity status using personally specific familiar faces and, therefore, ecologically valid face stimuli. It has been suggested that recognizing pictures of famous faces does not engage the normal face recognition processes that are engaged in the recognition of personally familiar faces (25–28). A specific familiar face indicates that the individual to whom the face belongs is encountered regularly, thereby the specific representation may be more flexible and more closely related to the feelings generated by the person. Indeed, our study shows that the feeling of familiarity in response to specific familiar faces is associated with a stronger emotional response than that in response to famous faces. The more homogeneous stimuli, the lack of an iconic image, or the more flexible representations may be responsible for this stronger emotional response (24). In previous studies (14, 24) involving healthy subjects, a decrease in SCRs and emotional neural activation, as measured in the paracingulate cortex, from unknown to famous and specific familiar faces was found with no difference between the famous and unknown faces. However, in a study by Ellis et al. (13), similarly to our study, the control subjects exhibited higher SCRs in response to the famous faces than those in response to the unknown faces, but our study showed that this effect was lower in response to the specific familiar faces. Hence, these results show that the response to famous faces is variable according to each study. Altogether, these results validate the hypothesis that famous materials are not adapted in familiarity studies. Finally, these findings shed light on the importance of emotion in specific familiar face processing compared to the processing of other types of faces (famous or unknown people). The processing of famous faces may be primarily based on basic or general representations, which would elicit more cognitive processes than emotional processes that are linked to the feeling of familiarity (29). If representations of famous people cannot be used by people with FD, these faces will activate generic representations that are, in fact, related to the people with FDs’ knowledge about the famous people. Therefore, the SCR level in response to famous faces is between that of unknown and familiar faces.

### A Continuum between Normal and Pathological; Familiarity

To the best of our knowledge, our study was the first controlled study to explore two groups of people with schizophrenia. Our results fit particularly well with the hypothesis of a continuum from normal to pathological functioning in FDs (9). Indeed, all people with schizophrenia exhibited lower SCRs, which has already been documented in the literature (16). Nevertheless, the patients with and without FDs exhibited different profiles of emotional responses to the faces, showing that their SCRs can still be modulated. The first stage of the disorder was captured by the FD− patients, who showed a global decrease in the feeling of familiarity as measured by their lower SCR amplitudes than those of the controls; however, the FD− patients still maintained differences in emotional feeling depending on the familiarity status. Thus, during this stage, the patients may have emotional disturbances, such as emotional flattering (30), as shown by the decreased SCR in people with schizophrenia without FDs, which may lead to a “weird” feeling in the presence others. Based on our results, we hypothesize that the lower feeling of familiarity in patients with schizophrenia may be a premise enabling the emergence of FDs as proposed by Coltheart et al. (31) in his two-step delusional models that explain the emergence of delusion. Therefore, it could be a necessary, albeit insufficient condition, for an FD to develop. During the second stage, the FD may be expressed in the FD+ patients, which, in turn, leads to an extinction of the feeling of familiarity. According to Hirstein’s model (1), the absence of the simultaneous activation of egocentric and allocentric representations of someone close to the patient may cause the feeling of a lack of familiarity and the subsequent decrease in the SCR observed in the patients with FDs. This model may also explain why the delusion of CS does not affect the response to famous people despite the absence of a feeling of familiarity. Notably, our results, which were based on the SCR, appear to be specific to the FD and independent of the medication doses or schizophrenia symptom severity.

Having a lower sense of familiarity implies a lower impression of the continuity of the world, which could then disrupt the sense of reality. Face-to-face encounters require responding and entering a relationship (which does not occur with famous people). Relationships with relatives involve the self, and familiar faces are embodied stimuli with meaning that include the self (27). The anomalies of “self-experience” are known to be specific to schizophrenia (32, 33). The sense of reality and the subsequent continuity of the world are obtained according to self-experiences. Hence, disturbances in the self-experience in schizophrenia could lead to a distorted sense of reality. Thus, from the phenomenological perspective, these disturbances could result in the emergence of schizophrenia (34). In a previous SCR study involving schizophrenic patients that did not consider FD (30), we used an equivalent paradigm with specific familiar, self and unknown faces. The people with schizophrenia exhibited the same familiarity in response to the self-face as that in response to the specific familiar faces. Therefore, a lower sense of familiarity in response to faces may involve a lower sense of self-face experience (as a part of face familiarity). From a phenomenological perspective, the disrupted sense of familiarity could, therefore, be a core feature of schizophrenia.

### Limitations of the Study

One limitation of this study is the absence of a standardized clinical evaluation of familiarity disorders. Nevertheless, to the best of our knowledge, no psychometric instrument has been validated to date. As an illustration of the need for a psychometric scale, the reported prevalence of Capgras syndrome in schizophrenic patients in the literature is very heterogeneous, ranging from 4.1% (35) to 28% (36) and up to 40% (37) in the same population (schizophrenia). A better categorization of FDs is a critical issue to be addressed by future studies.

Our control experiment using healthy subjects demonstrated a difference in attractiveness between famous faces and both specific familiar and unknown faces, while approachability was similar among the conditions. Moreover, a previous study showed that familiarity preference, which was evaluated by higher attractiveness, was preserved in schizophrenia (38). This difference is present only in famous faces. Thus, an effect of attractiveness on our primary results could lead to a greater SCR in response to famous faces (19). The control subjects exhibited higher SCRs in response to specific familiar faces than in response to famous faces. These data confirm that our study examined familiarity rather than attractiveness. In fact, the increased SCR in response to attractiveness appears to occur only in subliminal conditions (19).

Furthermore, a trend toward significance was observed for the difference between the patient groups in the positive PANSS subscores. This result is not surprising because FD+ patients have a delusion of familiarity that is rated in the positive PANSS subscore in items dedicated to delusion assessment. Nevertheless, the absence of a correlation between the positive PANSS subscore and the SCR amplitude excludes a direct relationship between this positive symptom and the observed SCR difference between the patient groups.

Finally, an individual’s mood has been shown to impact skin conductance (39). Nevertheless, mood is a tonic process that varies during the day by the minute and is more linked to tonic than phasic skin conductance, such as the SCR (39). Moreover, it has been shown that higher levels of both tonic and phasic SCRs are more linked to the negative symptoms (40, 41) because the negative symptoms could lead to the implementation of coping strategies in states of over arousal due to schizophrenia (42). In our study, the patients’ moods were not directly assessed; nevertheless, some items on the PANSS (such as the negative and general subscores) allow such an assessment, and no difference was observed between the groups; in addition, there was no correlation between the negative symptoms and the SCR results. Moreover, the absence of a difference in the SCRs among the three groups in response to the unknown faces suggests that the SCR could be the same in the control subjects and patients.

### Conclusion

To the best of our knowledge, this study is the first to compare patients with and without familiarity disorders using material that is specific to each participant to evaluate familiarity in schizophrenia. In the control subjects, the more familiar the person, the stronger the emotional response linked to the feeling of familiarity. The same process occurs in the FD− patients, although these patients display a weaker emotional response across all conditions as indicated by the lower SCR amplitudes. However, the FD+ patients experienced an absence of emotional responses linked to the feeling of familiarity regardless of the familiarity of the faces that were presented (known or unknown). The feeling of familiarity elicited by emotional processes is not modulated by the degree of familiarity of the perceived faces.

## Ethics Statement

The study was approved by the local ethics committee (CPP, Nord-Ouest IV, France), and written informed consent was obtained from each participant or guardian.

## Author Contributions

AA, AP, FD, GV, PT, and DP contributed to the conception or design of the work; AA and AP have made the acquisition of the data; AA, FD, and DP have analyzed and interpreted the data; AA drafted the work; AP, FD, GV, PT, and DP revising it critically for important intellectual content; and all authors have given their final approval of the version to be published and their agreement to be accountable for all aspects of the work in ensuring that questions related to the accuracy or integrity of any part of the work are appropriately investigated and resolved.

## Conflict of Interest Statement

The authors declare that the research was conducted in the absence of any commercial or financial relationships that could be construed as a potential conflict of interest. The reviewer YC and handling editor declared their shared affiliation.
